# Strategies towards attainment of universal coverage of long lasting insecticide treated nets (LLINs) distribution: experiences and lessons from Ghana

**DOI:** 10.1186/s13071-016-1328-5

**Published:** 2016-01-22

**Authors:** Kwame Gakpey, Aba Baffoe-Wilmot, Keziah Malm, Samuel Dadzie, Constance Bart-Plange

**Affiliations:** Ghana National Malaria Control Programme, Ministry of Health, Legon, Ghana; Noguchi Memorial Institute for Medical Research, P. O. Box LG 581, Legon, Ghana

## Abstract

Long lasting Insecticidal nets continue to provide effective protection against disease vectors including mosquitoes that transmit malaria. In many countries, promotion of LLIN usage and ownership has focused on the attainment of universal coverage. However modalities for achieving these objectives remain a challenge in many countries. This paper shares Ghana’s experience and strategies adopted to attain universal LLINs coverage.

## Letter

Many studies have shown the effectiveness of Insecticide Treated Nets (ITN) as an effective tool for preventing malaria transmission [1]. This is particularly the case since regular re-treatment of nets with insecticide has become unnecessary with the introduction of long-lasting insecticidal nets (LLIN) [2]. Recent efforts promoting the use of LLINs have shifted their emphasis from a focus on vulnerable populations to a broader objective of universal coverage, defined at the household level as the use of insecticide-treated nets by all household members regardless of age or gender [3]. There is an emerging consensus that a ratio of at least one LLIN for every two household members is typically sufficient to achieve universal coverage in a population [4].

Centralized mass distribution campaigns have served as the cornerstone of efforts to achieve universal coverage [5]. Recent evaluations of these campaigns support their effectiveness at broadening household ownership of LLIN, i.e. households with at least one LLIN increase substantially [6]. However, these campaigns at times fail to provide households with sufficient quantities of nets to reach the desired ratio of one LLIN per two household members [7, 8]. As a consequence, the effect of these campaigns on the proportion of all people sleeping under a LLIN often falls short of target levels.

In Ghana, the Ministry of Health (MOH)/Ghana Health Service (GHS) has promoted the use of LLINs and Indoor Residual Spraying (IRS) as proven malaria vector control interventions since 1998 and 2006 respectively. Long lasting insecticidal nets ownership and use have been low over the years. The overall objective among others was to attain 100 % households ownership of at least one ITN; 80 % of general population sleep under ITNs; 85 % children under-five and pregnant women sleep under ITN by the year 2015 [9].

The country had used different channels for the distribution of ITNs and mainly given to children under five years and pregnant women from 1998 to 2009. These channels included distribution through Maternal and Child Health Promotion campaigns, voucher schemes and highly subsidized sale of the nets at the health facilities among others. The use of the nets among children under five years of age and pregnant women was 28 % and 20 % respectively [10]. It was observed that many recipients of the ITNs did not use the nets because they did not know how to hang the nets.

In order, therefore, to overcome this barrier and also to achieve universal coverage, the nation in 2010 opted for the “Door-to-door distribution and Hang-Up of ITNs” whereby ITNs were not just given to household members but hanged in their sleeping places freely. This strategy has been implemented in all the ten regions of the country. The strategy involved the use of multi-sectoral Regional Committees with the Regional Ministers as the chairpersons. This was instituted in each region as the campaigns were rolled from one region after the other governed by the regions malaria prevalence rate and net availability. The committee’s terms of reference were to coordinate and oversee all regional activities for a successful implementation of the distribution and hang-up campaigns. The districts also formed similar committees which comprised the various stakeholders and notable among them were the District Health Management Teams, District Assemblies, the media, Ghana Education Service, Ministry of Agricultural, NGOs, chiefs and other traditional leaders, opinion and religious leaders and Unit Committee members.

This was followed by a Training of Trainers workshop organised at the regional level to train regional supervisors, district and sub-district health staff and Community Registration Assistants who registered all household members in the communities. Household registration data was collected and validated. Eighty percent (80 %) of the net needs were dispatched to the region and the remaining 20 % dispatched after the validation. Volunteers were given nails and ropes and orientation on proper ways of hanging bed nets and key messages such as airing of LLINs for 24 h or more before sleeping under them, sleeping under the LLINs every night, LLINs lasting for 20 washes when taken care of properly, washing dirty LLINs with mild soap and drying under shade, not washing LLINs in rivers or streams and not drying LLINs under the sun. Communities selected their hang-up volunteers and motivated them during the campaigns. Monitoring activities were conducted and weekly feedback meetings were held. The exercise was carried out by the MOH and GHS/National Malaria Control Programme (NMCP) in collaboration with the Department for International Development (DFID, the United Nations Children’s Fund (UNICEF), the Global Fund for ATM, the President’s Malaria Initiative/United State Agency for International Development (PMI/USAID), Malaria No More, ADRO, NET for Life, Humanity International, World Health Organization (WHO) and the World Bank.

The results of the strategy was that Twelve Million, Four Hundred and Eighty One Thousand, Three Hundred and Thirty Six (12,481,336) LLINs were distributed and hanged across the country from May 2010 to October 2012. This covered a population of 21,716,830 resulting in population coverage of 98 % well above the Millennium Development Goals for 2015. Total population registered for the exercise was 22,138,198. Although, no complimentary data on malaria reduction was carried alongside the campaign, a review of recent data from 2010 to 2012 [9] showed that malaria case facility rates (CFR) for under-fives has reduced by 53.9 % (1.3 to 0.6). The reduction may not be entirely due to the high coverage and usage of LLINs, however, we believe alongside other interventions being carried out in the country, the campaign may have contributed to the trend we observed.

Some of the lessons learnt were that some District Health Directorates in addition to support from their communities had financial support from their District Assemblies for the motivation of volunteers. Some communities contributed bowls of cocoa beans, plantain and other foodstuff to motivate the volunteers. Others in absence of cash or foodstuff organized clearing of farm lands for volunteers. Volunteer remuneration was at no cost to the national organizers. There was high level involvement of Assembly men, chiefs, and queen mothers who mobilized volunteers to hang the nets. Some community members used their private cars, donkeys, bicycles and motors to transport nets from the Sub-district to the communities. High levels of dedication, strong team work and collaboration were exhibited among the stakeholders such as Development Agencies, National Commission for Civic Education, Ghana Education Service and the Media. Social mobilization through the use of radio stations and community information systems made information readily available to community members and that contributed to the success of the hang-up campaign across the country. Indeed effective social mobilization was the bedrock of the campaign. We conclude that it is good to invest in malaria through the use of LLINs and malaria communication.

## Recommendations

We recommend that households that are given LLINs should be assisted to hang the nets around their sleeping places. This should be done with a strong partnership with the beneficiary communities, backed by mass media communication and social mobilization.
